# First Clinical Consensus and National Recommendations on Tracheostomized Children of the Brazilian Academy of Pediatric Otorhinolaryngology (ABOPe) and Brazilian Society of Pediatrics (SBP)^☆^^☆☆^

**DOI:** 10.1016/j.bjorl.2017.06.002

**Published:** 2017-06-27

**Authors:** Melissa A.G. Avelino, Rebecca Maunsell, Fabiana Cardoso Pereira Valera, José Faibes Lubianca Neto, Cláudia Schweiger, Carolina Sponchiado Miura, Vitor Guo Chen, Dayse Manrique, Raquel Oliveira, Fabiano Gavazzoni, Isabela Furtado de Mendonça Picinin, Paulo Bittencourt, Paulo Camargos, Fernanda Peixoto, Marcelo Barciela Brandão, Tania Maria Sih, Wilma Terezinha Anselmo-Lima

**Affiliations:** a Universidade Federal de São Paulo (UNIFESP), Escola Paulista de Medicina (EPM), São Paulo, SP, Brazil; b Universidade Federal de Goiás (UFG), Goiânia, GO, Brazil; c Pontifícia Universidade Católica de Goiás (PUC-GO), Goiânia, GO, Brazil; d Universidade Federal de Goiás (UFG), Hospital das Clínicas, Unidade de Cabeça e Pescoço, Goiânia, GO, Brazil; e Universidade Estadual de Campinas (UNICAMP), Faculdade de Ciências Médicas, Departamento de Oftalmo/Otorrinolaringologia, Campinas, SP, Brazil; f Universidade São Paulo (USP), Faculdade de Medicina de Ribeirão Preto, Departamento de Oftalmologia, Otorrinolaringologia e Cirurgia de Cabeça e Pescoço, Ribeirão Preto, SP, Brazil; g Universidade Federal de Ciências da Saúde de Porto Alegre (UFCSPA), Porto Alegre, RS, Brazil; h Hospital da Criança Santo Antônio, Serviço de Otorrinolaringologia Pediátrica, Porto Alegre, RS, Brazil; i Universidade Federal do Rio Grande do Sul (UFRGS), Programa de Pós-graduação em Saúde da Criança e do Adolescente, Porto Alegre, RS, Brazil; j Hospital de Clínicas de Porto Alegre, Porto Alegre, RS, Brazil; k Universidade São Paulo (USP), Hospital das Clínicas da Faculdade de Medicina de Ribeirão Preto, Ribeirão Preto, SP, Brazil; l Universidade Federal de São Paulo (UNIFESP), Escola Paulista de Medicina (EPM), Departamento de Otorrinolaringologia e Cirurgia de Cabeça e Pescoço, São Paulo, SP, Brazil; m Associação de Assistência a Criança Deficiente (AACD), Clínica de Otorrinolaringologia, São Paulo, SP, Brazil; n Universidade Federal do Ceará (UFC), Fortaleza, CE, Brazil; o Hospital Pequeno Príncipe, Curitiba, PR, Brazil; p Serviço de Assistência Integral à Criança Traqueostomizada (SAIT), Belo Horizonte, MG, Brazil; q Universidade José do Rosário Vellano (UNIFENAS), Curso de Medicina, Belo Horizonte, MG, Brazil; r Universidade Federal de Minas Gerais (UFMG), Hospital das Clínicas (HC), Pneumologia Pediátrica, Belo Horizonte, MG, Brazil; s Universidade Federal de Goiás (UFG), Unidade de Terapia Intensiva, Goiânia, GO, Brazil; t Universidade Estadual de Campinas (UNICAMP), Faculdade de Ciências Médicas, Departamento de Pediatria, Campinas, SP, Brazil; u Universidade de São Paulo (FMUSP), Faculdade de Medicina, São Paulo, SP, Brazil; v International Society for Otitis Media (ISOM), São Paulo, SP, Brazil; w Universidade São Paulo (USP), Faculdade de Medicina de Ribeirão Preto, Ribeirão Preto, SP, Brazil

**Keywords:** Tracheostomy, Child, Guidelines, Consensus, Traqueostomia, Criança, Diretrizes, Consenso

## Abstract

**Introduction:**

Tracheostomy is a procedure that can be performed in any age group, including children under 1 year of age. Unfortunately health professionals in Brazil have great difficulty dealing with this condition due to the lack of standard care orientation.

**Objective:**

This clinical consensus by Academia Brasileira de Otorrinolaringologia Pediátrica (ABOPe) and Sociedade Brasileira de Pediatria (SBP) aims to generate national recommendations on the care concerning tracheostomized children.

**Methods:**

A group of experts experienced in pediatric tracheostomy (otorhinolaryngologists, intensive care pediatricians, endoscopists, and pediatric pulmonologists) were selected, taking into account the different regions of Brazil and following inclusion and exclusion criteria.

**Results:**

The results generated from this document were based on the agreement of the majority of participants regarding the indications, type of cannula, surgical techniques, care, and general guidelines and decannulation.

**Conclusion:**

These guidelines can be used as directives for a wide range of health professionals across the country that deal with tracheostomized children.

## Resumo

**Introdução:**

A traqueostomia é um procedimento que pode ser realizado em qualquer faixa etária, inclusive em crianças abaixo de um ano. Infelizmente no Brasil existe uma enorme dificuldade dos profissionais de saúde em lidar com esta condição e uma falta de padronização dos cuidados.

**Objetivo:**

Este consenso clínico realizado pela Academia Brasileira de Otorrinolaringologia Pediátrica (ABOPe) e Sociedade Brasileira de Pediatria (SBP) tem como objetivo gerar recomendações nacionais sobre os cuidados e condutas diante das crianças traqueostomizadas.

**Método:**

Foram selecionados um grupo de especialistas com experiência em traqueostomia na infância (otorrinolaringologistas, pediatras intensivistas, endoscopistas, pneumopediatras) que tivessem comprovada atuação prática no assunto, e que também contemplassem as diversas regiões do Brasil, de acordo com os critérios de inclusão e exclusão.

**Resultados:**

Os resultados gerados neste documento foram a partir da concordância da maioria dos participantes em relação as indicações, tipo de cânula, técnicas cirúrgicas, cuidados e orientações gerais e decanulação.

**Conclusão:**

Estas diretrizes poderão servir como norteadoras para os mais diversos profissionais de saúde em todo país que lidam com as dificuldades das crianças traqueostomizadas.

## Introduction

The international literature traditionally reports the need for tracheostomy in approximately 0.5–2% of children undergoing intubation and mechanical ventilation in intensive care units. In the last 60 years, changes in the epidemiology of infectious diseases and the evolution of medical techniques have changed the indications for tracheostomy. Tracheostomy can be performed in children of any age group, including infants under 1-year of age. The increase in the use of tracheostomy in this age group has been attributed to the greater survival of preterm infants and those requiring prolonged ventilation.^1^ This procedure, when performed in children and especially infants and newborns, has been associated with greater morbidity and mortality when compared to adults.^2^, ^3^

Regarding the indications of tracheostomy in childhood, we have seen significant changes in recent years.^4^, ^5^

It can be currently observed that in Brazil huge difficulty for health professionals to deal with this condition is apparent, as well as the lack of care standardization. At the worldwide level, this problem has been attenuated in other countries in recent years, through the discussion and suggestion of consensuses among professionals who have contact with tracheostomized children.^6^, ^7^, ^8^, ^9^

The creation of specific care teams for these children has shown to optimize care and can potentially reduce not only the hospital costs but also the suffering of the child and the family involved, promoting improved care with resolute perspectives.^10^ Some studies in the literature have depicted the difficulties and the negative impact for the child, parents and/or caregivers in the presence of tracheostomy in childhood.^11^, ^12^

In a review paper published in 2016 on tracheostomy complications,^13^ compared the last 3 decades, the presence of granuloma, infection and cannula obstruction are still among the most frequent problems, that is, the ones that depend on care and guidelines. Unfortunately, there are yet no lines of care for that matter in our country.

The mortality rates related to tracheostomy in children range from 0% to 5.9% in the international literature.^13^ A Brazilian study carried out in Porto Alegre in 2009 reported a mortality rate of 4%.^14^

In Brazil, this lack of care standardization is due to the lack of national guidelines to guide the Brazilian Unified Health System (SUS – *Serviço Único de Saúde*) and the National Health Agency (ANS – *Agência Nacional de Saúde*), which is reflected in the lack of availability of material needed to care for these patients, such as tracheostomy cannulas in the medical care services, as well as the lack of training of the medical and non-medical teams that treat these young patients. Without proper training for cannula replacement, these children are maintained in the Tertiary System for these procedures, without assistance or basic guidelines. Unfortunately, even in the major centers and Tertiary Hospitals, these basic materials (tracheostomy tubes for regular change) are not available, so these children belong to the system, but there are no codes in SUS that contemplate performing this procedure, which is essential for the child not to develop emergency situations, such as airway obstruction or hospital admission due to lung infections. Therefore, after being tracheostomized, these children are often lost in the public system, as there is no adequate flow of referral and follow-up, as it occurs with other life-threatening diseases.

In 2016, the Brazilian Academy of Pediatric Otorhinolaryngology (ABOPe – *Academia Brasileira de Otorrinolaringologia Pediátrica*) during its most important national scientific event, launched the project to carry out a National Consensus for recommendations in tracheostomized children. Thus, the aim of this Consensus is to obtain the opinion of a group of experts in tracheostomized children, aiming to establish national guidelines for medical conducts and care, allowing in the near future the creation of “standards of care” and the creation of a flowchart for the referral and treatment of these children in the Unified Health System.

## Methods

During a meeting, we requested colleagues from different regions of the country to create a group of experts on the subject. We also requested the opinion of colleagues who were not present, but who had acknowledged practical experience in this area and were interested in participating in this project.

Two initial coordinators, with proven practical experience in Pediatric Airways in National University Institutions, in addition to scientific activities in the subject, received indications for the selection of the group to be created. The Brazilian Society of Pediatrics was approached and invited to provide support and partnership in this work, and it indicated members who were pediatricians and experienced in the care of tracheostomized children to participate in the consensus.

*Inclusion criteria for selection of the expert group*

- Declared interest in participating in the Consensus;
- Otorhinolaryngologists with proven experience in pediatric airways, either through consistent publications on the subject, or by practicing in referral services in the country;
- Otorhinolaryngologists who were not present at the meeting, but who the coordinators considered important regarding their performance in the area in our country;
- Group of pediatricians designated by the Brazilian Society of Pediatrics: intensivists, bronchoscopists, pulmonologists with proven experience in the management of tracheostomized children.

*Exclusion criteria*

- Not having expressed an interest in participating after the invitation;
- Another representative from the same service was already participating in the Consensus;
- Not having broad performance or continuous clinical practice with tracheostomized children.

After an extensive review of the subject by all consensus members with indicated literature and after requesting reviews of specific topics for each participant, a questionnaire was prepared to be answered in person without prior disclosure to the participants. A questionnaire containing 43 multiple-choice questions was discussed. Those questions for which there was an agreement of more than 50% of participants regarding the answer were considered as a consensus. When none of the answers obtained a 50% agreement, the results were presented for each question and the considerations were discussed by the group. Participants were asked to answer based on what they considered ideal, even though they might not necessarily be able to practice that in their services due to financial and logistical limitations. Questions on surgical techniques were answered only by participating surgeons.

The questions were divided into topics: tracheostomy indications, type of cannula, surgical technique, care, guidance, and decannulation.

During the consensus, the need to create guidelines related to daily practice concerning phonatory valve use, complications, speech therapy indication, and attendance at school activities or at daycare centers was also raised.

## Results

### Indications

It was the consensus among the members that tracheostomy in children should be performed in a surgical center. The Consensus members considered it essential to perform a preoperative endoscopic airway evaluation (EAE) before the tracheostomy to assess the causes of respiratory obstruction and based on the findings and the future therapeutic proposal, decide the best location for the tracheostomy. In cases where it is impossible to perform this examination prior to the tracheostomy, it is suggested that it be performed after the procedure to verify the adequate tracheostomy positioning, for the follow-up of the treatment of airway pathologies, to guide future treatments, as well as to report the presence of a patent airway above the tracheostomy. Airway endoscopy is understood as the examination from the nasal cavities to the main bronchi, making it possible to evaluate all possible points of upper airway obstruction. The examination with flexible and rigid endoscopes should be performed whenever possible. Ideally, airway endoscopy should be performed in a surgical center under anesthesia in spontaneous ventilation, for greater safety and opportunity to address eventual pathologies during the same examination.

If the endoscopic evaluation is not possible at the time of the tracheostomy, the consensus members considered it should be indicated as early as possible, ideally up to 15 days after the tracheostomy, and no later than 30 days after the procedure. This indication is absolute, considering that the effect on the inflammatory process in its acute phase can modify the prognosis, particularly in the case of stenotic lesions with scarring of the larynx.

In the case of children in an intensive care unit (ICU) with extubation failure due to high respiratory obstruction, the members considered the indication of airway endoscopy to be absolute in the following situations:

- after the second elective extubation failure and/or;
- in the persistence of stridor or dysphonia after 72 h of extubation.

**Figure 1:**
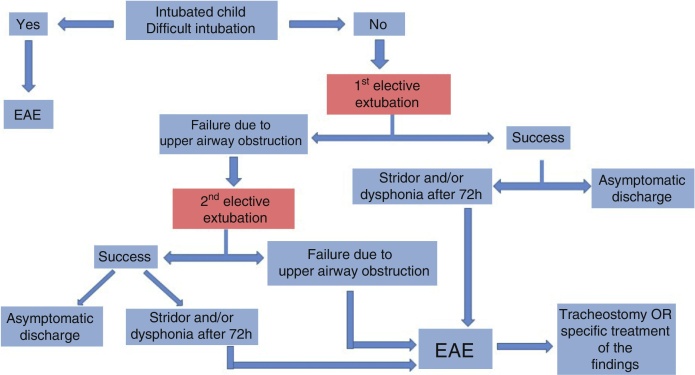
Flowchart indicating preoperative endoscopic airway evaluation (EAE) in the intubated child.

In the case of children with a history of difficult intubation, airway endoscopy is suggested before the first elective extubation (Fig. 1).

The tracheostomy indications should guide the type of cannula and ventilatory support required, as well as follow-up and therapeutic and decannulation planning. All these variables and perspectives should be discussed with the family since the indication of the tracheostomy.

### Cannula types

**Figure 2:**
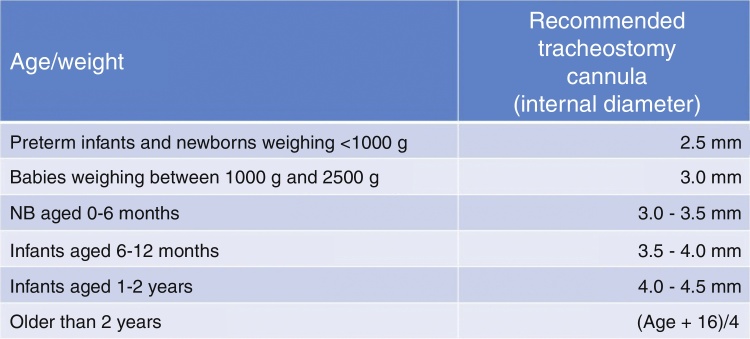
Diameter of tracheostomy cannula appropriate for age/weight. The number of the tracheostomy cannula corresponds to the internal diameter in millimeters (mm). NB, newborn; g, grams.

The sizes of the cannula should be adequate for the child's weight and age (Fig. 2 – cannula sizes), and the use of cuffs is indicated only to optimize ventilation and to temporarily reduce the impact of aspiration, when present.

When indicated, the use of cuff pressure should be measured and maintained at the maximum up to 20 cm H20 or 15 mmHg.

The use of biocompatible siliconized or plastic tracheostomy cannulas (Table 1) is recommended. The use of metal cannulas is not recommended in children due to their low biocompatibility, lack of malleability and greater risk of tracheal injury, since children, unlike adults, do not restrict their cervical and body movements when they submitted to a tracheostomy. The adaptation of cannulas with inadequate lengths is not recommended either. The easy cleansing of the internal mandrel creates a false sense of low obstruction risk, which has not been demonstrated by evidence, and can delay the exchange of the cannulas. Furthermore, it is necessary to consider that the internal mandrel reduces the cannula lumen and can lead to respiratory failure despite the supposedly adequate size for the age.

**Table 1:** Description of the tracheostomy cannulas most frequently found in the national market with composition material and durability indicated in the package insert by the manufacturer.

Canulla/brands	Material	Durability
SHILEY	Siliconized PVC	28 days – cleansing and reuse are not recommended.
PORTEX	PVC	29 days – cleansing and reuse are not recommended.
BIVONA	Silicone	9 months with cleansing every 30 days
TRACHOE	Siliconized PVC	120 days
COMPER^a^	PVC	30 days

a Attention regarding the dimensions of this cannula, of which length is on average 7 mm longer than the others.

### Surgical technique

The surgical technique used by surgeons should vary according to the age group and the pathology. Even considering these variables, all surgeons use nonabsorbable repair sutures or stoma maturation with absorbable sutures fixing the trachea to the skin depending on the patient's age and characteristics, or the characteristics and requirements of the intensive care unit. Another variable discussion point according to the patient's pathology is the height of the tracheostomy, which will depend on the diagnosis and the future treatment for decannulation.

It was a consensus among the authors that the first change should occur after one week of the tracheostomy, when the nonabsorbable sutures should also be removed to prevent exacerbation of the local inflammatory processes. The use of antibiotic prophylaxis is recommended, and a first-generation cephalosporin is usually used. The indication of chest X-ray after tracheostomy should be considered in children under one year of age, to certify that the cannula is well-positioned in relation to the carina if there is no intraoperative endoscopy confirmation. In older children, the postoperative chest X-ray may be standardized in some intensive care units, but it is not routinely recommended by the group.

There was no consensus on the need to fix the tracheostomy cannula to the skin; however, 44.4% of the participating surgeons reported using this fixation. In turn, 33.3% of surgeons fix the tracheostomy cannula to the skin in the first week only in special circumstances; and the others do not think it necessary to perform this fixation, using only the cord.

### Care and recommendations

#### Aspiration

Advice on aspiration technique, need for humidification, and regular cannula changes before discharge is always recommended. It is imperative that surgeons who perform the tracheostomy provide these recommendations themselves, even if there is a multiprofessional team capable of training the caregivers.

The aspiration is recommended depending on the amount and characteristic of the tracheal secretion observed in the child, without pre-established schedules. It is recommended that caregivers perform the aspiration at least at waking and before bedtime. The list of minimum materials available at home should include: non-sterile gloves, single-use disposable aspiration catheters, and a tracheostomy cannula half-size smaller than the one being used. A modified clean technique, as defined by the American Thoracic Society (ATS) is recommended for cannula handling, aspiration and change of dressing and cord: non-sterile gloves, but sterile aspiration catheters.

The aspiration technique can show slight variations, but in general, it should be gentle yet efficient. Attention should be given to:

- choice of catheter size, not exceeding two-thirds of the size of the cannula;
- depth of aspiration to prevent trauma to the trachea distal to the cannula tip;
- aspiration time to prevent hypoxia, pneumothorax, vagal reflexes.

Regarding the type of cannula fixation, there is no evidence to recommend any preference between cord and Velcro. There was a consensus among the experts that no specific type of aspirator has proven superior efficiency.

**Figure 3:**
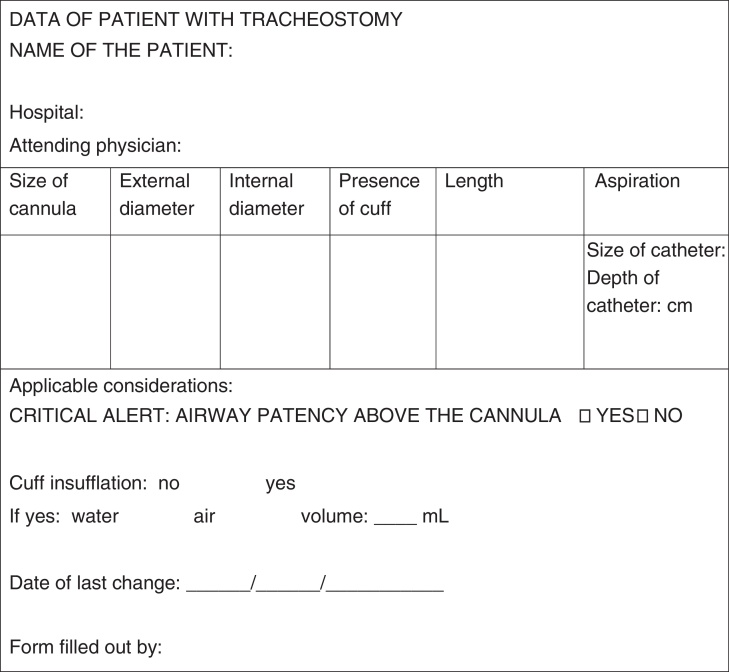
ID card of the tracheostomized child (CICT).

It is recommended that the services provide a tracheostomized child with an identification card at the time of hospital discharge (Fig. 3), which should include the following information (card template):

- name and age of the child and date of tracheostomy;
- critical alert specifying whether the airway above the tracheostomy is patent or not;
- size of the cannula being used and recommended aspiration catheter number;
- recommended aspiration depth;
- identification of the hospital or referral service that follows the child and the attending physician.

**Figure 4:**
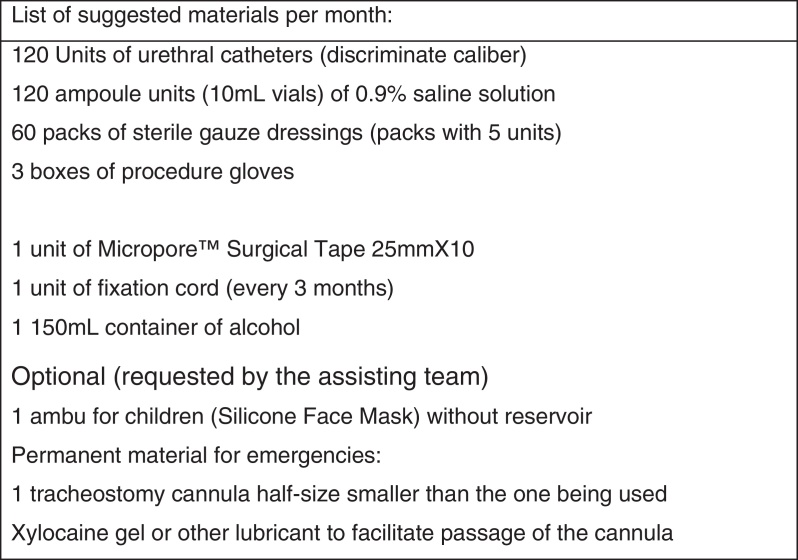
Monthly list of materials.

A list of basic materials that should be available for care and emergency situations is also suggested (Fig. 4).

##### Cannula changes and care of the stoma

Sporadic changes of tracheostomy cannulas must comply with the manufacturer's instructions found in the package insert of the cannula being used, and should not exceed one month. In some cases, it is possible to sanitize and reuse the cannula; however, this practice should be indicated by the manufacturer. Metallic cannulas should not be used in children, under any circumstances. Changes must be performed by qualified and trained professionals. It is recommended that the attending physician teach the caregivers to perform cannula replacement. Whether the cannula change will be performed at home, in a hospital environment or under anesthesia will depend on the team's experience and the characteristics of the patient and his/her airway. Case-by-case evaluation is recommended.

In case of accidental decannulation, a cannula of the same size or a smaller one should be used for replacement. If these are unavailable, we recommend the use of a half-smaller size tracheal tube, followed by the immediate referral of the child to the reference service for tracheostomy cannula repositioning. The group also emphasizes the importance of teaching parents about the risk of decannulation, and of demonstrating the child's position (cervical hyperextension) for this repositioning, as well as stoma exposure, by moving away the skin from the region.

Stoma hygiene once daily is recommended, or more frequently, depending on the climatic conditions and the child's general health status, presence of excessive secretions or local complications. The routine use of ointments is not recommended, except in the presence of signs of inflammation of the peristomal skin. In the case of ointment use, it should always follow medical prescription. The use of gauze pads between the cannula and the skin of the neck is also debatable, and it is a consensus that the most important effort would be to prevent the accumulation of moisture in the peristomal skin region. Sometimes the use of gauze pads can promote the accumulation and retention of secretions and peristomal moisture, and thus, if used, gauze pads should be changed whenever they are soiled.

Establishing standardized protocols would reduce the risk of complications, particularly those related to the occurrence of peristomal inflammatory processes. Creating an emergency checklist could reduce the risk of the most feared complication of all, i.e., accidental decannulation (Fig. 4).

##### Inhalation, nebulization, and humidification

The use of inhalation and nebulization should follow medical guidelines, and the practice of inhalation with physiological solutions and its benefit aiming at humidifying the airway is not well established. Aiming to keep the airway hydrated and humidified, it is always necessary to recommend that the child maintain an adequate water intake, considering the greater fluid loss due to the tracheostomy.

The use of hydroscopic filters or “false nose” may be useful in most cases and their indication should be individualized in each case, depending on the amount of secretion and, especially, the child's lung function.

##### Secretion culture

The tracheostomy is an area colonized by microorganisms. The collection of tracheal secretions would be indicated only in cases of tracheitis with hospitalization indication or in the preoperative period of laryngotracheal reconstruction surgeries to guide intravenous antibiotic therapy. It is worth remembering that the diagnosis of tracheitis is based on clinical criteria: increase and/or alteration in tracheal secretions associated or not with fever, worsening of the general health status and tachycardia in the presence of a normal chest X-ray. The culture of tracheal secretion is indicated to guide the antibiotic therapy in severe cases, for which hospitalization is required.

##### Phonatory valves

The use of phonatory valves is recommended both to facilitate communication and speech development, and perhaps mainly to reduce the risk of bronchoaspiration, promoting the return of subglottic pressure. The use of phonatory valves is contraindicated in cases of:

- Severe airway stenosis;
- Need to use cannula with an insufflated cuff;
- Severe tracheomalacia;
- Restrictive pulmonary disease;
- Severe neurological disorder or comatose patient.

Some situations may hinder, but not prevent the use of the phonatory valve and, therefore, each case should be evaluated individually. The valve should be indicated by the team that follows the tracheotomized patient, with the endorsement of the attending physician.

### Complications

Persistent trachea-cutaneous fistulas after decannulation are considered minor complications of tracheostomies. If they persist after three months, medical evaluation for surgical closure should be indicated.

Granulomas are quite frequent complications of tracheostomies and may occur externally in the stoma, or internally in the trachea. For external granulomas, in addition to the use of topical medications, the reinforcement in local care is indicated. Internal granulomas should be approached when obstructive, and especially during the decannulation process, even when there is partial obstruction.

### Speech therapy and audiological evaluation

Speech therapy and audiological evaluation have been recommended in tracheostomized children in the prelingual period for communication development and in cases of dysphagia.

### Decannulation

The following were considered factors that contraindicate decannulation:

- Absence of EAE;
- Dependence on mechanical ventilation in the last 3 months;
- Dependence on tracheostomy for pulmonary toilet.

After detailed and complete evaluation of the airway while the child is awake and under anesthesia on spontaneous ventilation, the following decannulation protocol is suggested for children older than 2 years:

- Progressive reduction in cannula size;
- Cannula occlusion during the day at home;
- 68.75% of the authors believe that nocturnal occlusion of the cannula should be carried out only in a hospital environment;
- Decannulation of patients with comorbidities should be carried out at the intensive care unit in the first 24 h;
- Observation in hospital environment for at least 48 h after decannulation.

For children under 2 years the recommended protocol is:

- The cannula occlusion period prior to decannulation is not necessary;
- Observation during the first 24 h after decannulation in the ICU regardless of comorbidities;
- Observation in hospital environment for at least 72 h after decannulation.

Performing a polysomnography with an occluded cannula, although recommended by some services, was not recommended by the group. The performance of airway endoscopy while the child is under induced sleep and observation of the respiratory pattern during the examination and also in the hospital was considered sufficient to rule out the presence of obstruction that prevents decannulation.

#### Access to school

The presence of tracheostomy alone should not prevent the child from attending school. However, it is necessary that a qualified person provide the necessary care, including cannula aspiration and clearing, if necessary.

## Final considerations

This consensus was created aiming to generate national guidelines by specialists in relation to the medical practices and care of tracheostomized children, but it is noteworthy to emphasize that it does not mean they must be followed exactly as they were portrayed here. We acknowledge the diversity and limitations of our vast country, but we also deem it extremely important that our public entities pay special attention to this group of children, who, as they have a tracheostomy, become so vulnerable.

## Conflicts of interest

The authors declare no conflicts of interest.
